# Error in Funding/Support

**DOI:** 10.1001/jamahealthforum.2025.2413

**Published:** 2025-06-13

In the Viewpoint titled “Achieving Racial and Ethnic Equity in COVID-19 Vaccination: From Individual Readiness to Health System Readiness,”^1^ published online on July 30, 2021, in *JAMA Health Forum*, the grant award number under Funding/Support was incorrect. The Viewpoint was corrected.
